# Continuous Synthesis of Carbon‐Coated Na_3_V_2_(PO_4_)_3_ by Segmented Flow Tubular Reactor

**DOI:** 10.1002/smtd.202502388

**Published:** 2026-03-24

**Authors:** Samuel Franz Gatti, Anton Beiersdorfer, Ionut Mihalcea, Sigita Trabesinger, Andrea Testino

**Affiliations:** ^1^ Center For Energy and Environmental Sciences Paul Scherrer Institute (PSI) Switzerland; ^2^ École Polytechnique Fédérale de Lausanne (EPFL) Switzerland

**Keywords:** continuous synthesis, NVP, SFTR, sodium ion batteries

## Abstract

Scaling‐up the synthesis of materials via hydrothermal and solvothermal routes is a long process and usually involves time‐ and cost‐intensive parameter optimization. The solution to this challenge is continuous synthetic approaches. In this study, we present an easily scalable, continuous synthesis approach for the sodium ion battery cathode material Na_3_V_2_(PO_4_)_3_ (NVP) using a segmented flow tubular reactor (SFTR) following a polyol synthesis route, followed by a high‐temperature treatment to form a carbon coated active material. We investigate the phase formation of the SFTR‐derived material during high‐temperature treatment through in situ XRD. We investigate the material morphology‐structure–performance relationship to understand the consequences of this synthesis and processing methodology in comparison to conventional hydrothermal synthesis methods. Finally, the electrochemical evaluation in half‐cell configurations demonstrates that the SFTR‐derived NVP exhibits excellent performance, with a low capacity fading of 0.016% per cycle at 1C and excellent high‐rate capability up to 10C. The presented synthesis methodology is readily transferable to other phosphate‐based chemistries. Furthermore, these results underscore the potential of continuous synthesis methods to accelerate the scalable production of high‐performance battery materials.

## Graphical abstract

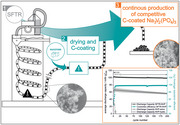

## 1 Introduction

The global energy crisis rapidly demands new energy‐storage materials at scale. In the context of energy transition and world‐wide decarbonization, scalability and reproducibility of materials synthesis in combination with fast assessment at the prototype level is a fundamental need to discover new and to optimize existing materials [1]. Both new and known materials with improved functional properties due to optimizations of particle size and morphologies may be synthesized via bottom‐up wet‐chemistry routes. However, laboratory‐scale synthesis methods typically rely on small batch reactors, resulting in a limited amount of the material of interest. Mass and energy transfer, and in particular optimal heating rates during synthesis, strongly depend on the reactor volume. Therefore, increasing the amount of product by increasing the reactor volume leads to a loss of control over material properties. This is of particular relevance for nanosized materials: the lack of reliable synthesis methods for upscaling is a major obstacle for their development and assessment at a relatively large scale [2, 3, 4].

**FIGURE 1:**
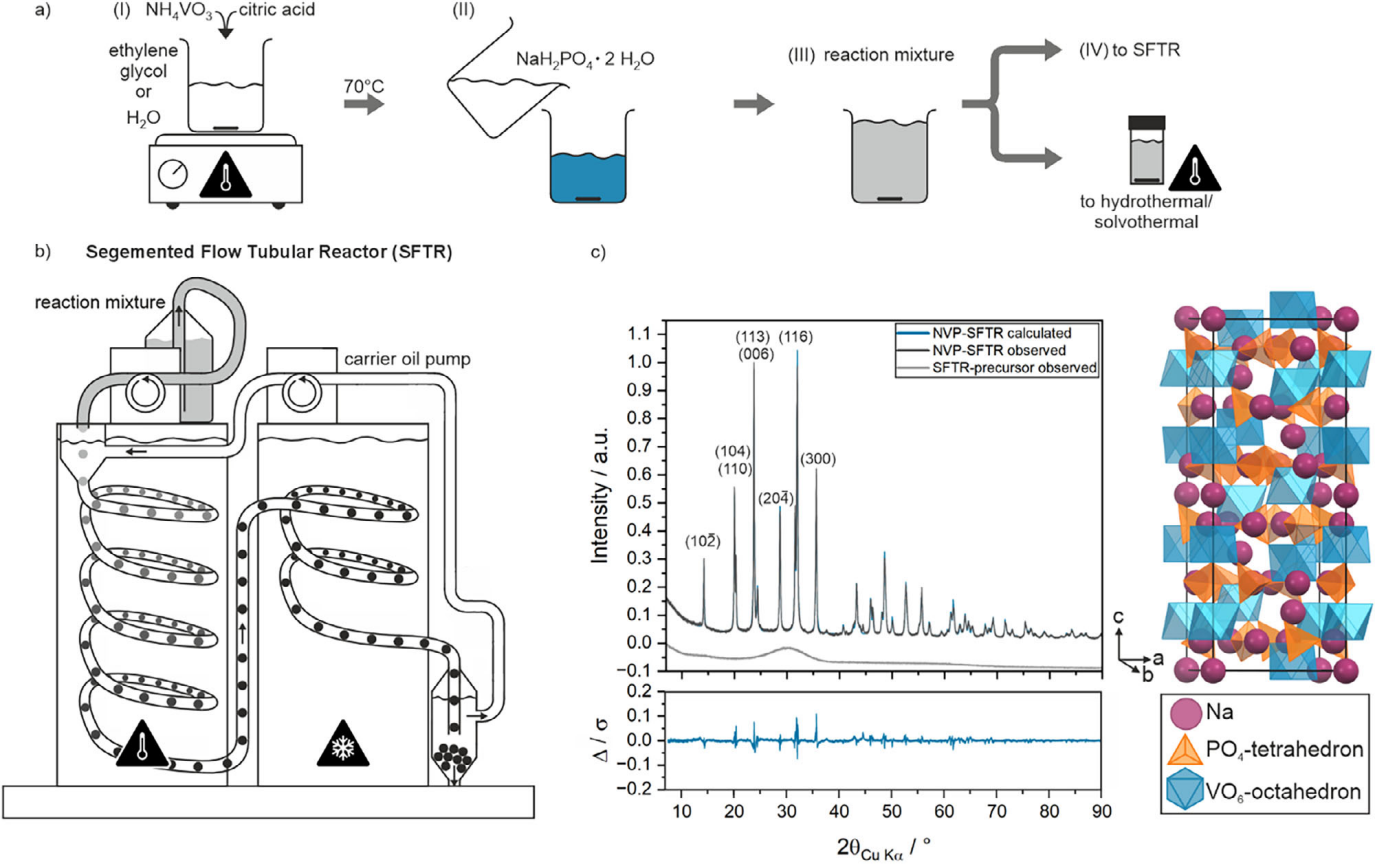
Schematic illustration of the individual steps (I‐IV) for the reaction mixture preparation (a) and schematic illustration of the SFTR working principle (b). Two HPLC pumps are used for either the reaction mixture (here: Na_2_HPO_4_ ·  H_2_O + NH_4_VO_3_ in EG) whereas a gear pump is used for the carrier oil (here: Dodecane). XRD patterns of the SFTR‐precursor (light grey), after the high‐temperature treatment (NVP‐SFTR, black), and results of Rietveld refinement (blue) with the corresponding residual error (blue) and refined NVP structure (c).

A solution to this problem requires high degrees of particle size control, with simultaneous high material throughput, ideally in a continuous manner. Segmented flow tubular reactors (SFTR) offer these features. In an SFTR, individual droplets of the reaction mixture in a solvent (A) are segmented using a secondary immiscible solvent (B). The segmented fluid is pumped in a tube and kept at a defined temperature for the entire reaction time by a tempered bath. A schematic representation of the SFTR concept is given in Figure 1b. Because of the segmentation, ideal plug‐flow (which assumes the absence of axial mixing due to the parabolic flow commonly attained in unsegmented tubular reactors) and no contact between the reaction mixture and the inner wall of the pipe (due to the carrier oil protective film, which prevents fouling), is ensured [4]. Therefore, this reactor design follows the same performance equations, which relate input and output in a chemical reactor, as hydrothermal/solvothermal batch reactors, hence high productivities can be achieved [5]. Due to the small reaction volume, the droplets are rapidly heated, achieving 99% of the bath temperature in approximately 10 s [6]. When nucleation and growth reactions of solid matter are under kinetic control, a rapid increase of temperature results in a rapid increase of nucleation rate, and, therefore, in small particle sizes and narrow particle size distributions. This allows the SFTR to tune particle sizes effectively and produce small particles with narrow particle size distributions. Since each droplet is a micro reactor, subjected to identical mass and energy transport, the particles produced in each droplet have equivalent properties and morphologies, and therefore large quantities can be achieved over time in continuous mode without product quality loss [6, 7, 8]. In contrast to conventional hydrothermal or solvothermal synthesis routes, SFTR offers the advantage of continuous production of material and eliminates practical batch‐process derived issues, such as batch‐to‐batch variations of material properties and the need for autoclaves, which come at high capital expenditure at scale, thus offers economic advantages [9, 10]. Other common alternatives to hydrothermal synthesis, for instance, solvothermal synthesis that does not require high pressures due to the use of high‐boiling‐point organic solvents and therefore may not require autoclaves, but at an increased operational expenditure due to the use of more expensive solvents and the need for waste management. Nevertheless, in the intermediate scale range, where materials for feasibility studies and prototyping are needed in the range of 100–1000 g, low‐cost and high‐boiling‐point organic solvents, such as ethylene glycol (EG), may offer a trade‐off. Additionally, the tunability of the scale‐out rather than scale‐up approach of the SFTR reactor concept avoids expensive and time‐consuming trial‐and‐error parametrization phases required for scaling up hydrothermal processes [11]. This makes SFTR synthesis a promising candidate for scale‐up nanoparticle production in the intermediate production range, which has been proven in the past for metal‐oxide and metal nanoparticle scale‐up synthesis [4, 6, 7, 8].

As a case study of effective upscaling of a relevant material for sodium ion battery production, we present our results on continuous synthesis of Na_3_V_2_(PO_4_)_3_ (NVP). NVP exhibits the NASICON‐type structure, which allows for fast sodium‐diffusion, and therefore high rate‐capability at a moderate capacity of 117.6 mAh g^−1^. However, the low electronic conductivity requires small particle sizes (≤800 nm) [12] to maintain high rate capability, as well as high‐control over particle morphology [13, 14]. Due to this, NVP is typically synthesized through hydrothermal batch synthesis, with the aforementioned scale‐up limitations [13, 15]. To further improve the electronic transport of the material, carbon coatings are applied. Typical reaction products of such batch‐processes are crystalline NVP, which require an additional high‐temperature treatment to form a conductive carbon coating [12, 13].

In this work, we demonstrate the feasibility of SFTR synthesis for phosphate‐based materials, using carbon‐coated NVP as a model system. Through in situ XRD and coupled TGA‐MS, we investigate the phase‐evolution and decomposition processes during high‐temperature treatment. The electrochemical performance of the obtained material is evaluated in a half‐cell configuration and compared to batch‐synthesized counterparts. Our findings highlight the potential of SFTR as a scalable, reproducible, and economically viable route for producing high‐performance battery materials, with broader applicability to other phosphate‐based chemistries such as LiFePO_4_ (LFP) and Li(FexMn_1__−__x_)PO_4_ (LMFP).

## 2 Experimental

### 2.1 NVP Synthesis

For NVP synthesis, NH_4_VO_3_ (*AlfaAesar*, >  99%), citric acid monohydrate (Sigma–Aldrich, ≥99.0%), ethylene glycol (EG) (Sigma–Aldrich, ≥99.0%), NaH_2_PO_4_ · 2 H_2_O (Sigma–Aldrich, ≥99.0%), *n*‐Dodecane (*n*‐C_12_) (Sigma–Aldrich, ≥99.0%), sucrose (*Fluka*, ≥ 99.0%) were used without further purification.

A mixture of NH_4_VO_3_ (0.10 m) and citric acid monohydrate (0.05 m) in EG was heated to 70°C for 30 min to pre‐reduce V^5+^. The obtained dark‐blue solution was cooled down to room temperature. A second solution, NaH_2_PO_4_ · 2 H_2_O (0.15 m) in EG, was added to achieve a final solution with stoichiometric ratios of Na : V: P of 3 : 1: 3 (see Figure 1a, I‐IV). The final solution was fed at a flow rate of 11.5 mL min^−1^ using HPLC pumps into a segmented flow tubular reactor (SFTR, schematic illustration in Figure 1b, polyfluorinated alcohol (PFA) flow tube Ø 3 mm) with the thermostatic bath temperature set to 165°C. A SFTR reactor features a flow tube, that carries the reaction mixture, which is segmented into droplets by a segmenting fluid which is heated in a temperature bath. *n*‐C_12_ was used as the segmenting fluid (due to its immiscibility with EG) at a flow rate of 70 mL min^−1^, controlled by a gear pump. The reaction time (residence time in the tubular reactors’ high‐temperature bath) for each droplet was 6.2 min before quenching it in the second thermostatic bath, which was set to room temperature.

The two immiscible phases, EG with the produced solid and *n*‐C_12_, were separated and the oil recirculated. The EG phase was centrifuged, decanted, and residual EG was removed at 80°C at reduced pressure. The production yield was 4.9 g of NVP‐precursor per hour of reactor operation. Hereafter, the material obtained at this step is referred to as SFTR‐precursor. The obtained SFTR‐precursor was mixed with sucrose in a ratio of 1: 0.35 by weight, suspended in EG, and ultrasonicated for 10 min (97.5 W pulse for 5 s, 20 s break) using a *Vibra‐Cell* (*Sonics*) ultrasonicator. EG was removed at 80°C under reduced pressure.

In order to obtain final crystalline NVP, the precursor was thermally treated under 5% H_2_ in Ar in a tubular furnace (*Carbolite*), first heating at 5°C min^−1^ to 350°C with a 3 h dwell time, then heating at 5°C min^−1^ up to 800°C with a 16 h dwell time and followed by natural cooling, as reported in the literature [14]. The carbon‐coated material, obtained after the thermal treatment, is further referred to as NVP‐SFTR. For comparison with conventional synthesis methods, two samples were synthesized applying the same parameters as reported in the literature [13], but using a larger stirred hydrothermal/solvothermal reactor (*4760 Series* (450 mL), *Parr Instruments*), followed by the same high‐temperature treatment protocol as used for the SFTR‐NVP.

The hydrothermal and solvothermal syntheses (hereafter: Hydro‐precursor/Solvo‐precursor before the thermal treatment and NVP‐hydro/NVP‐solvo after the thermal treatment) were performed using the same precursor solutions described for SFTR synthesis, however, at higher concentrations as reported in Table 1. A reaction time of 16 h at 180°C was used, as described in the literature [13]. A summary on the conditions applied for SFTR‐, hydrothermal, and solvothermal precursor synthesis is shown in Table 1.

**TABLE 1:** Synthesis conditions of SFTR‐precursor (after pre‐reduction of V^5+^), Hydro‐precursor and Solvo‐precursor.

	Concentrations (in reactor)			Ageing	Reaction	Sol‐	Reaction	Pressure
	NH_4_VO_3_	NaH_2_PO_4_ · 2 H_2_O	H_3_Cit · H_2_O	time	volume	vent	temperature	
SFTR‐precursor	0.033 m	0.100 m	0.017 m	6.2 min	11.5 mL min^−1^	EG	165°C	1.0 bar
Hydro‐precursor	0.150 m	0.225 m	0.075 m	16 h	160 mL	H_2_O	180°C	10.0 bar
Solvo‐precursor	0.150 m	0.225 m	0.075 m	16 h	160 mL	EG	180°C	1.5 bar

### 2.2 Characterization

Powder X‐ray diffraction (XRD) patterns were collected using a *Bruker D8 Advance* diffractometer with a Cu Kα radiation source from 7° to 90° at 0.007°/step. Crystalline domain sizes were calculated from XRD patterns using the *Rietveld* method for full‐pattern refinements embedded the GSAS‐II software package [16]. The instrumental broadening contribution was calibrated using a LaB_6_ standard. Thermogravimetric analysis (TGA) to determine the C‐content was performed on a *Mettler Toledo TGA/DSC1* at a heating rate of +5°C min^−1^ in air. To monitor the sucrose decomposition during high‐temperature treatment, Ar‐atmosphere was used instead and a similar high‐temperature protocol to the synthesis conditions was used.

To monitor the crystallization and carbonization reaction during high‐temperature treatment, in situ XRD on pressed pellets (∅13 mm, 300 mg, 1 mm in thickness) was conducted on a *Panalytical Empyrean* equipped with a high‐temperature furnace *HTK1200N* (*Anton Parr) and* Cu Kα radiation source. Due to the high scattering‐cross section of Ar, N_2_ atmosphere was used. Patterns were recorded for 10 min every 50°C in the 2θ range from 19° to 33.5° at 0.0167°/step, starting from 50°C. A heating rate of +5°C min^−1^ was applied. Additionally, a 3 h hold step at 350°C was added in order to mimic synthesis methodology. Due to time constraints of experiments, the hold step at 800°C was shortened to 8 h (from the 16 h used during synthesis). Afterward, the mixture was cooled down to RT at −10°C min^−1^. The heating program is shown in Figure 3a, left panel.

Surface area and pore size distribution were determined using Brunauer–Emmett–Teller (BET) analysis and N_2_ gas sorption was performed on a *Quantachrome Instruments Autosorb iQ XR*. The true density of the material was obtained using He‐pycnometry on a *Micromeritics AccuPyc II 1340* pycnometer. Particle size distribution was measured on a *Horiba LA‐950* particle size analyzer. Scanning electron microscopy (SEM) micrographs were acquired using a *Zeiss Ultra 55*. High‐resolution transmission electron microscopy (HR‐TEM) was performed on a *Jeol NeoARM* at 200 kV.

### 2.3 Electrochemical Testing

For electrochemical tests, coin‐cell‐like PSI standard cells (as described in ref. [17]) with working electrodes containing carbon coated NVP (4.3–4.5 mg · cm^−2^), Super P (*Timcal*), and polyvinylidene fluoride (PVDF, *Sigma*) in a ratio of 8 : 1 : 1 by weight, and metallic Na counter electrodes (*Xiamen AOT Electronics Technology*) were assembled, using 1 m NaPF_6_ in dimethoxyethane (DME, *E‐lyte*) as an electrolyte. The electrodes were prepared by slurry coating (wet thickness 150 µm) on an Al‐current collector before drying at 120°C under reduced pressure. For long‐term stability evaluation, tests were performed on *LBT20084* (*Arbin Instruments*) cycler with 5 formation cycles at C/20 followed by long‐term performance tracking at 1C (1C = 117.6 mA g^−1^). Rate tests were performed between C/20 and 10C. For each of the reported discharge capacities, at least 3 individual cells were averaged. Cyclic voltammetry was performed at sweep rates of 5, 10, 50, 100 and 5 µV s^−1^ from 2.6 to 3.7 V using a MPG‐2 battery cycler (*Biologic*).

## 3 Results and Discussion

### 3.1 Synthesis and Characterization

NVP precursors were synthesized via two approaches: a segmented flow tubular reactor (SFTR) and conventional hydrothermal/solvothermal batch methods adapted from the literature [13]. Following precursor synthesis, a carbon coating was applied using sucrose, followed by high‐temperature annealing. The high‐temperature step is thereby inevitable for successful NVP‐synthesis to form a carbon‐coating on the surface of the particles, which is required due to the inherent low electronic conductivity of the material, regardless of the crystallinity of the NVP precursor [18].

The SFTR process yielded 4.9 g of NVP precursor per hour per reaction tube, while the batch hydrothermal/solvothermal method yielded 7.3 g per batch, corresponding to 0.45 g per hour. This highlights the significantly higher productivity of the SFTR approach. According to XRD, the initial SFTR‐precursor remains amorphous, as shown in Figure 1c. However, after mixing with sucrose and following high‐temperature treatment at 800°C, the material crystallizes in the rhombohedral *R* $\bar{3}$ *c* space group with crystallite sizes of 62.9 nm according to *Rietveld* refinements (Figure 1c). The high‐temperature step results in a final yield of 4.6 g (92.7% of theoretical yield with respect to vanadium) of carbon coated NVP‐SFTR per tube and hour of reactor operation. This corresponds to 4.2 g of NVP per hour of reactor operation, which is a significant increase in yield, comparing to the batch‐processed hydrothermal/solvothermal syntheses, which yields about 0.45 g of NVP per hour of reactor operation.

**FIGURE 2:**
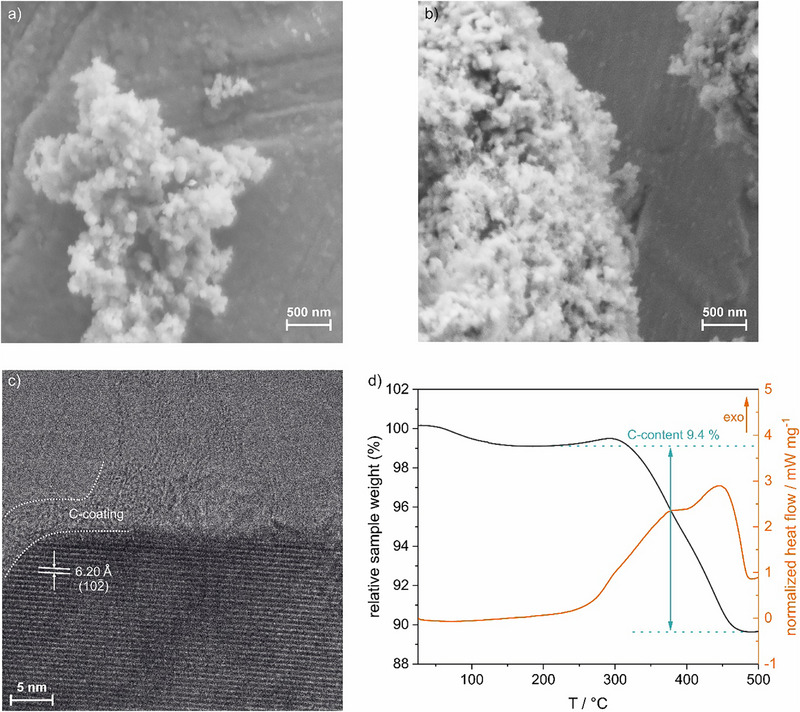
SEM Micrographs of (a) the SFTR‐precursor and (b) NVP‐SFTR after high‐temperature treatment. (c) HR‐TEM image of NVP‐SFTR. (d) TGA of NVP‐SFTR in air for C‐content quantification.

After the high‐temperature treatment, the initial small particle size of the SFTR‐precursor is maintained, as shown in SEM images (Figure 2a,b). The particle size, observed by SEM, is well in agreement with crystalline domain sizes obtained from *Rietveld* refinements (for refinement parameters see Table S1), however, these primary particles agglomerate to form secondary particles > 1 µm (see SEM image in Figure 2b and particle size distribution in Figure S2). Without sufficient amounts of sucrose, primary particles exceeding 1 µm in diameter are formed (see SEM image in Figure S3). Based on that we conclude that the amount of carbon and the carbon source plays an important role during the high‐temperature treatment, to prevent sintering/coagulation of the material. This is in line with work by Mizuno et al., indicating a strong effect of the carbon source on morphology and electrochemical performance [19]. To quantify the carbon content, TGA analysis was conducted (see Figure 2d). A final carbon content of 9.4 wt.% was determined, corresponding to a total yield of 32.6 wt.% of carbon per g of sucrose, and resulting in a high specific‐surface area of 54 m^2^ g^−1^ (see Figure S4). However, as can be seen in the HR‐TEM image in Figure 2c, the synthesis methodology results in a non‐conformal carbon coating with thickness up to 5 nm for NVP‐SFTR and a highly crystalline NVP material.

### 3.2 In Situ Investigation of High‐Temperature Treatment

**FIGURE 3:**
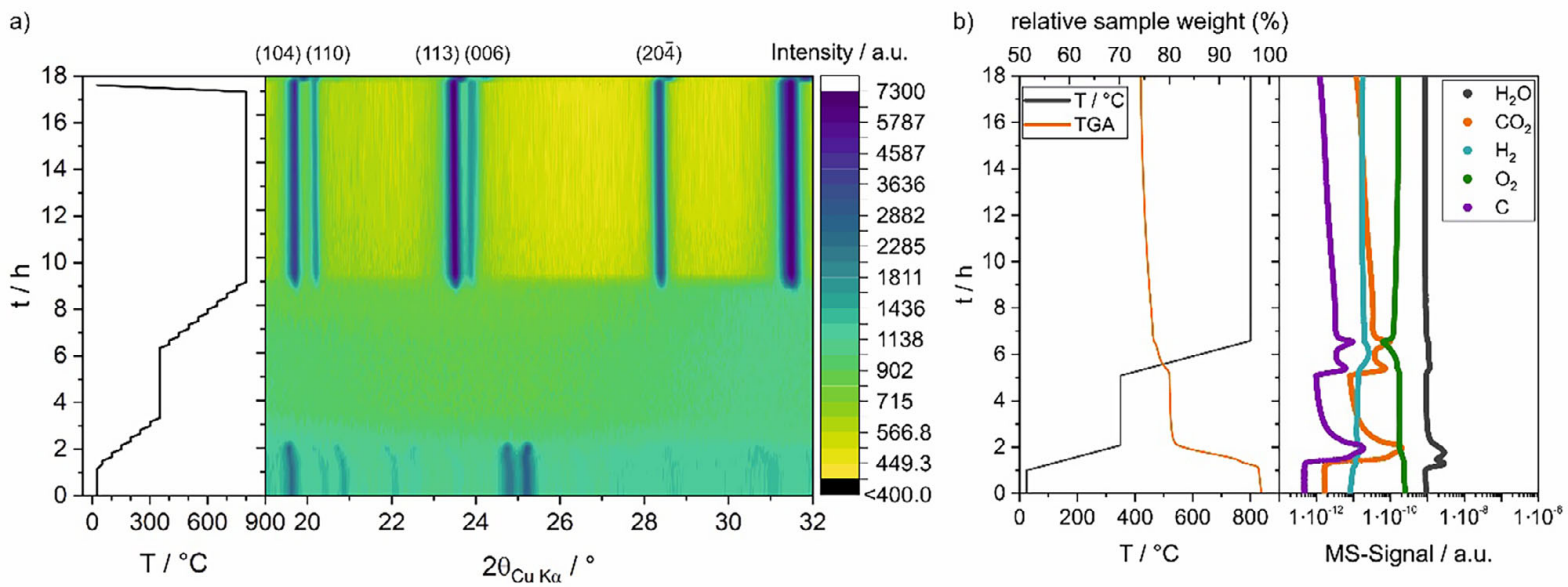
In situ high‐temperature XRD in N_2_ atmosphere (a) and TGA‐MS in Ar atmosphere (b) of the SFTR‐precursor mixed with sucrose.

With the aim to understand the processes during the high‐temperature treatment in more detail, an in situ XRD and TGA experiment, using similar heating and cooling parameters as the synthesis conditions, were performed. An XRD contour map visualizes the temperature‐dependent structural changes, as shown in Figure 3a, while individual patterns during the experiment are provided in Figure S5. In the initial state, a high background is observed similar to the one observed for the SFTR‐precursor before mixing with sucrose in Figure 1c, and several reflections, corresponding to crystalline sucrose (see XRD patterns in Figure S5a). The high background and amorphous bump at 30 ° 2θ confirm, that the amorphous SFTR‐precursor is maintained during the mixing step with sucrose. The sucrose reflections shift toward lower angles between RT and 150°C, indicating the thermal expansion of the unit cell. Between 150°C and 200°C these reflections disappear, which is well in agreement with the melting point of sucrose, however, the high background remains, suggesting that this temperature does not affect the amorphous NVP‐precursor. During the holding step at 350°C, as well as during the temperature ramp to 650°C, no significant change can be observed in XRD patterns. Finally, between 650°C and 700°C, the SFTR‐precursor starts to crystallize and form crystalline NVP in the rhombohedral *R* $\bar{3}$ *c* space group. Simultaneously, the background level is significantly reduced, indicating that the amorphous phase reacts and is converted into the crystalline one. Upon further increase in temperature to 800°C, the reflections shift to lower angles, corresponding to the thermal expansion of the NVP unit cell. During the dwell time at 800°C, only small changes can be observed, as shown in the XRD patterns in Figure S5b. The fast crystallization reaction already at 700°C implies that the crystallization time and temperature could be potentially reduced for leaner and more efficient NVP production. After cooling down to RT, all characteristic reflections of rhombohedral NVP can be observed, while a shift of all reflections toward the higher angles indicate thermal contraction of the unit cell.

To gain insights into the carbonization process of sucrose and eventual additional decomposition processes, TGA‐MS was performed to obtain complementary data to the in situ XRD experiment during the high‐temperature treatment of the amorphous SFTR‐precursor after mixing with sucrose, as shown in Figure 3b. A first mass‐loss of 5.8% is observed between 100°C and 150°C, with a simultaneous increase in the H_2_O and CO_2_ signal, which is most likely due to the release of volatile small organic molecules and H_2_O adsorbed on the surface of the material. At higher temperatures, between 150°C and 350°C, a mass loss of 12.2% and corresponding CO_2_ and H_2_O evolution are observed, indicating decomposition reactions of organic compounds. This is accompanied by an endothermic event (see TGA and DSC signal in Figure S6), which in combination with in situ XRD results indicate a melting of sucrose, followed by thermal decomposition of sucrose. This reinforces the necessity of the low‐temperature step at 350°C, where (1) melting of sucrose and homogenization of the sample are occurring, and (2) where the decomposition of sucrose to form a carbon coating is initialized. However, the synthesis procedure should be further optimized by decreasing the temperature of this low‐temperature step to adjust this temperature to just above the melting point of sucrose, allowing for homogenization before the rapid decomposition reaction, as the violent decomposition reaction already takes place before reaching 350°C.

Between 350°C and 600°C, there is neither mass loss nor evidence for gaseous species on the mass‐spectrometry signal, while between 600°C and 700°C, an additional mass‐loss of 3.2%, accompanied by CO_2_ release and O_2_ uptake is observed. However, the O_2_ uptake is an artifact of the measurement due to residual O_2_ in the Ar‐flow of the TGA furnace. At the same time, the fact, that the *m/z*  =  12 (C) and *m/z*  = 44 (CO_2_) signals show an identical trend, suggests that no additional C‐containing organics are released from the sample, since they would yield in additional C‐signal, which does not show up in the CO_2_ signal. This implies, that no volatile organic species are formed in the sample and hence, we assume that the SFTR‐precursor does not contain any remaining solvents or other organics.

**FIGURE 4:**
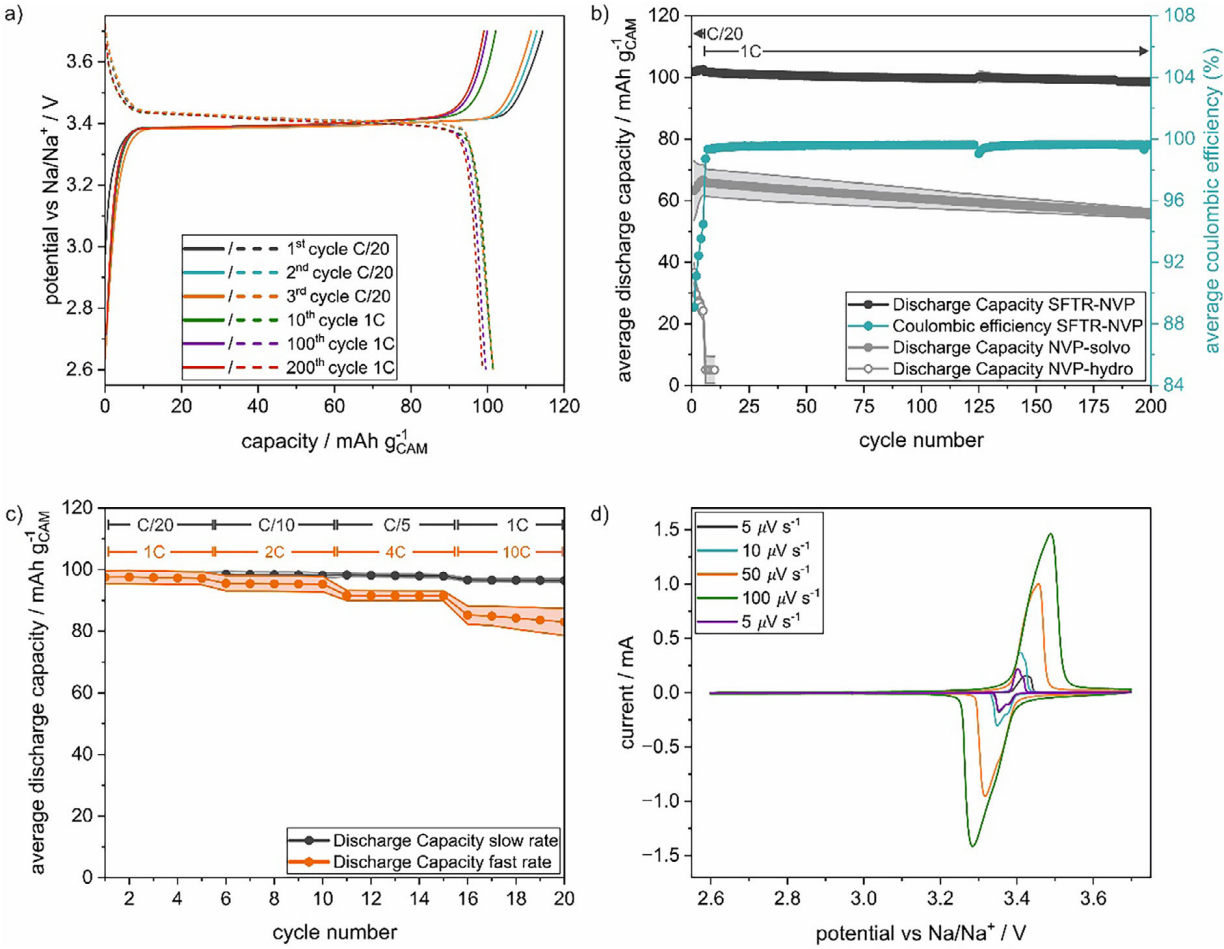
(a) Voltage curves of long‐term cycling cell for NVP‐SFTR. (b) Averaged capacity retention for NVP‐SFTR, NVP‐hydro and NVP‐solvo, as well as coulombic efficiency for NVP‐SFTR of long‐term cycling cells. (c) Rate test at high and low rates for NVP‐SFTR. (d) Cyclic voltammogram at different sweep rates for NVP‐SFTR.

Furthermore, assuming, that the total mass‐loss (see TGA in Figure 3b), observed during the high‐temperature treatment, originates only from sucrose decomposition, this translates into a residual of 10.5% of sucrose‐derived mass in the final material, which is well in agreement with the detected carbon content in the final product (see Figure 4a). Therefore, the observed decomposition reactions during TGA‐MS measurements (Figure 3b) are highly likely related to the sucrose decomposition, which shows that no additional mass‐loss is derived from the SFTR‐precursor, and thus there are no remaining organics after the drying procedure. This further confirms the in situ XRD findings, that the SFTR‐precursor only starts to react at elevated temperatures above 600°C.

### 3.3 Electrochemical Evaluation

Electrochemical performance was tested to evaluate the quality and suitability of NVP‐SFTR for battery applications. As can be seen in Figure 4a,b, at the rate of 1C, NVP‐SFTR shows a discharge capacity retention of 96.9(1) % over 200 cycles (not including the formation cycles at C/20) in a half‐cell configuration, with low polarization. During extended cycling, electrolyte decomposition and anode degradation may impact the cycle life, hence, long‐term cycling was limited to 200 cycles. This equals to a capacity loss of 0.016% per cycle, and therefore an expected lifetime to 80% SOH of > 1000 cycles (assuming constant fading rates), despite the high surface area of nano‐sized NVP‐SFTR. The capacity retention is comparable to literature reports, which have investigated different synthesis parameters (temperature, pH, precursors) based on hydrothermal and sol–gel synthesis routes, resulting in a variety of particle sizes and particle morphologies [13, 14, 20]. Therefore, the SFTR synthesis methodology produces a competitive material to conventional lab‐scale methods. However, as shown in Figure 4b, in comparison to the high initial capacity of 101.9(11) mAh g^−1^ of NVP‐SFTR, scaled‐up hydrothermal (NVP‐hydro) and solvothermal (NVP‐solvo) synthesis in EG (reproduced from literature [13]) without re‐parametrization for the increased reactor volume results in poor cyclability and initial discharge capacities of 37(3) and 63(3) mAh g^−1^, respectively. We attribute the lack of capacity to the formation of partially non‐coated single crystals exceeding 3 µm in particle size, as shown in SEM images, provided in Figure S7. This is in line with the decrease in capacity for NVP particles exceeding 800 nm without carbon coating reported by Jiang et al. [12] The formation of large particles in the scaled‐up hydrothermal synthesis showcases the difficulties of upscaling using conventional hydrothermal/solvothermal methods, which can be overcome using SFTR‐synthesis.

A full comparison of particle size, electrochemical parameters, and material yields per hour of reactor operation and solvent volume used for the different synthesis procedures is shown in Table 2.

**TABLE 2:** Comparison of different properties and yield for NVP‐SFTR, NVP‐hydro, and NVP‐solvo after thermal treatment.

Material	Primary particle size^a^	Average initial discharge capacity at C/20 / mAh g_NVP_^−1^	Average capacity retention after 200 cycles at 1C (%)	Reactor yield / g h^−1^ L_solv_ ^#^ ^−1^
NVP‐SFTR	100–200 nm	102(3)	96.9(1)	6.92
NVP‐hydro	> 1 µm	37(3)	0.0^***^	3.13
NVP‐solvo	150–250 nm^**^	63(3)	84.7(1)	3.13

a* estimated from SEM images, see Figure 2b for NVP‐SFTR, **Fehler! Verweisquelle konnte nicht gefunden werden**. (a) for NVP‐solvo and (b) for NVP‐hydro.

** platelets, thickness 10–30 nm.

*** for NVP‐hydro: cells stopped after 10 cycles due to cell‐failure.

^#^ solv = solvent used for the reaction. solv = EG for NVP‐SFTR and NVP‐solvo and H_2_O for NVP‐hydro.

Rate tests were performed for NVP‐SFTR in the range of C/20 to 10C. To minimize aging effects, separate cells were assembled for “slow rates” below 1C and “high rates” above 1C (Figure 4c). Average discharge capacities of 105.9(2), 102.1(9), 100.2(3) and 97.1(5) mAh g^−1^ were observed after 5 cycles at C/20, C/10, C/5 and 1C, respectively. At higher rates, we observed excellent rate capabilities with average discharge capacities of 98(2), 96 ± 2, 96(2) and 85(4) mAh g^−1^ after 5 cycles at 1C, 2C, 4C and 10C at areal loadings of 4.3–4.5 mg_NVP_ cm^−2^. This corresponds to a capacity retention of 80.0% at 10C with respect to C/20, which is similar to lab‐scale sol–gel and solvothermal derived NVP at comparably high areal loadings [12, 21, 22]. The high‐rate capability is in agreement with the low polarization observed in the cyclic voltammogram (Figure 4d), which additionally shows a small peak splitting of both, oxidation and reduction peaks. This might be due to the different sodium sites (6‐ and 8‐fold coordinated), whereas a low potential difference of the two different sites indicates high ionic conductivity [15]. Overall, the SFTR synthesis route offers a scalable and reproducible (see Figure S8) alternative to conventional batch methods, producing NVP with competitive electrochemical performance compared to optimized hydrothermal and sol–gel methods reported in literature [12, 13, 14, 20, 21, 22]. In summary, SFTR‐derived NVP achieves similar or superior rate capability and cycle life in comparison to up‐scaled hydrothermal/solvothermal routes, while enabling continuous production and higher yields.

## 4 Conclusions

In this study, we have demonstrated the successful continuous synthesis of carbon‐coated Na_3_V_2_(PO_4_)_3_ (NVP) using a segmented flow tubular reactor (SFTR), offering a scalable and reproducible alternative to conventional hydrothermal and solvothermal methods. The SFTR process enables precise control over particle size and morphology through rapid heating and short reaction times, resulting in nano‐sized NVP particles with narrow size distributions and high crystallinity after thermal treatment.

We investigated the ongoing processes during the high‐temperature treatment through a combination of in situ XRD and TGA‐MS. We conclude that the carbon source has a crucial role during the high‐temperature treatment by preventing sintering of the material, maintaining the small initial particle size. Therefore, the high‐temperature step is necessary for both the NVP crystallization, but also the formation of a conductive carbon network. Based on these findings, we suggest the following adjustments of the synthesis procedure: (1) decrease the temperature of the initial hold step at 350°C to just above the sucrose melting point for improved homogenization, (2) decrease the time of this step significantly and (3) decrease the time for the final step at 800°C significantly.

Electrochemical testing of the SFTR‐derived NVP revealed competitive performance in comparison to literature reports and excellent long‐term cycling stability, with only 0.016% capacity fading per cycle at 1C, and high rate capability up to 10C, even at practical areal loadings [12, 13, 14, 20, 21, 22]. In contrast, scaled‐up hydrothermal and solvothermal syntheses, without re‐optimization, resulted in significantly lower capacities and poor cycling performance due to uncontrolled particle growth and insufficient carbon coating.

These findings highlight the advantages of SFTR synthesis in overcoming the scalability and reproducibility challenges associated with traditional batch processes. The method not only improves yield and material quality but also enables continuous production, making it highly suitable for intermediate‐scale manufacturing. Furthermore, the approach is readily transferable to other phosphate‐based cathode materials such as LiFePO_4_ (LFP) and Li(Fe_x_Mn_1__−__x_)PO_4_ (LMFP), supporting broader applications in next‐generation sodium‐ and lithium‐ion batteries.

## Conflicts of Interest

The authors declare no conflicts of interest.

## Supporting information




**Supporting File**: smtd70624‐sup‐0001‐SuppMat.docx.

## Data Availability

The data that support the findings of this study are available from the corresponding author upon reasonable request.
