# Supplementary material for: Continuous Synthesis of Carbon‐Coated Na3V2(PO4)3 by Segmented Flow Tubular Reactor
Source: Small Methods. 2026 Mar 24;10(9):e02388. doi: 10.1002/smtd.202502388 (PMC13159419; doi:10.1002/smtd.202502388)
Supplement: Supplementary file 1 — Supporting File: smtd70624‐sup‐0001‐SuppMat.docx. [file SMTD-10-e02388-s001.docx]

**Continuous Synthesis of Carbon-Coated Na_3_V_2_(PO_4_)_3_ by Segmented Flow Tubular Reactor**

Samuel Franz Gatti^1,2^, Anton Beiersdorfer^1^, Ionut Mihalcea^1^, Sigita Trabesinger^1^ and Andrea Testino^1,2,*^

^1^Center for Energy and Environmental Sciences, Paul Scherrer Institute (PSI), Switzerland

^2^École Polytechnique Fédérale de Lausanne (EPFL), Switzerland

*andrea.testino@psi.ch

Keywords: continuous synthesis, NVP, sodium ion batteries, SFTR

**Supporting Information**

1. **Supplementary data**

**1.1 Structural analysis**

The XRD powder pattern of SFTR-NVP after high-temperature treatment was refined in the *R*$\overline{3}$*c* space group (**a = 8.72560(1)** $Å$**, c = 21.8389(1)** $Å$**, V = 1440.18(1)** $Å^{3}$, $\chi^{2}$ = 6.35; ICSD collection code 248140).

Supplementary Table 1. Structural parameters of high-temperature annealed NVP-SFTR, assuming negligible microstrain.

| Atom | x/a | y/b | z/c | Occupancy | Coordination Number | *Wyckoff* site |
| --- | --- | --- | --- | --- | --- | --- |
| V | 1/3 | 2/3 | 0.01865 | 1.00 | 8 | 12c |
| Na1 | 1/3 | 2/3 | 0.16670 | 0.80 | 6 | 6b |
| Na2 | 2/3 | 0.96966 | 0.08330 | 0.73 | 8 | 18e |
| P | 0.9555 | 1/3 | 0.08330 | 1.00 | 4 | 18e |
| O1 | 0.1415 | 0.50340 | 0.08045 | 1.00 | 6 | 36f |
| O2 | 0.5428 | 0.84682 | 0.97445 | 1.00 | 4 | 36f |


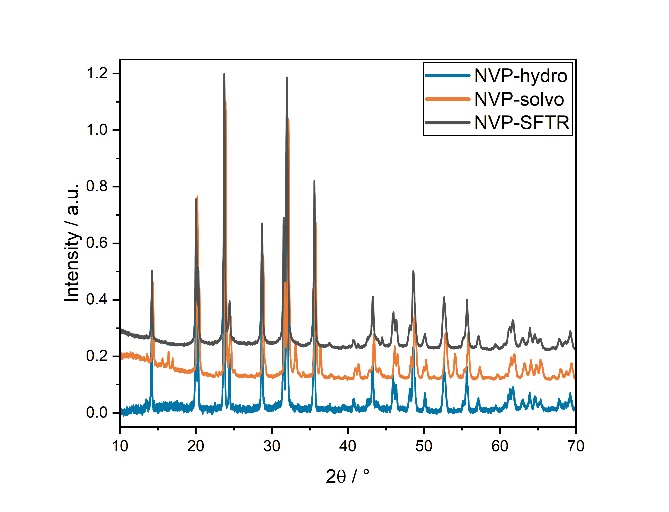


Supplementary Figure 1: Comparison of powder XRD patterns of NVP-SFTR, NVP-hydro and NVP‑solvo

**1.2 Particle size distribution**

Supplementary Figure 2 shows particle size distributions obtained by dynamic light scattering (DLS) in H_2_O. The material was ultrasonicated for 15 min., however, due to the strong agglomeration of the particles introduced by the hydrophobic C-coating predominantly agglomerates and agglomerates of agglomerates are observed in the particle size distribution. Hence, the particle size distribution shows significantly larger particle diameters with respect to SEM and XRD. Nevertheless, the particle size distribution of agglomerated particles agrees with SEM observations.


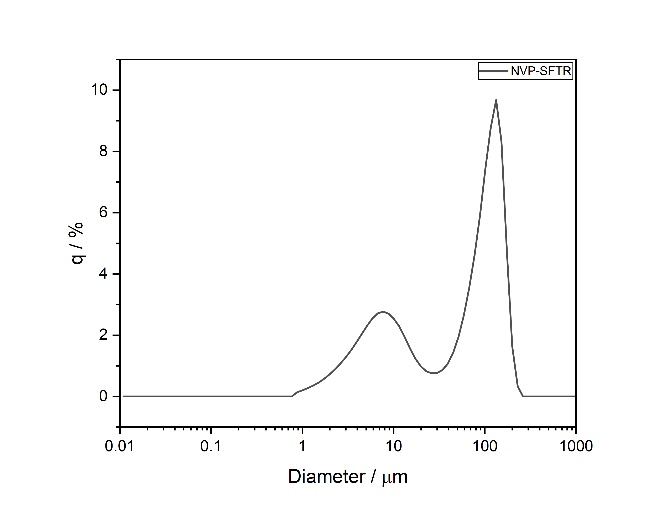


Supplementary Figure 2: Particle size distribution of NVP-SFTR after high-temperature treatment

**1.3 High-temperature treatment information**

The carbon source was observed to not only result in a porous, conductive network in the final material, but also to prevent sintering to form large particles during the high-temperature treatment step, as shown in Supplementary Figure 3. With in-sufficient amount of carbon-precursor (sucrose) large single crystals form (Supplementary Figure 3 (a)).


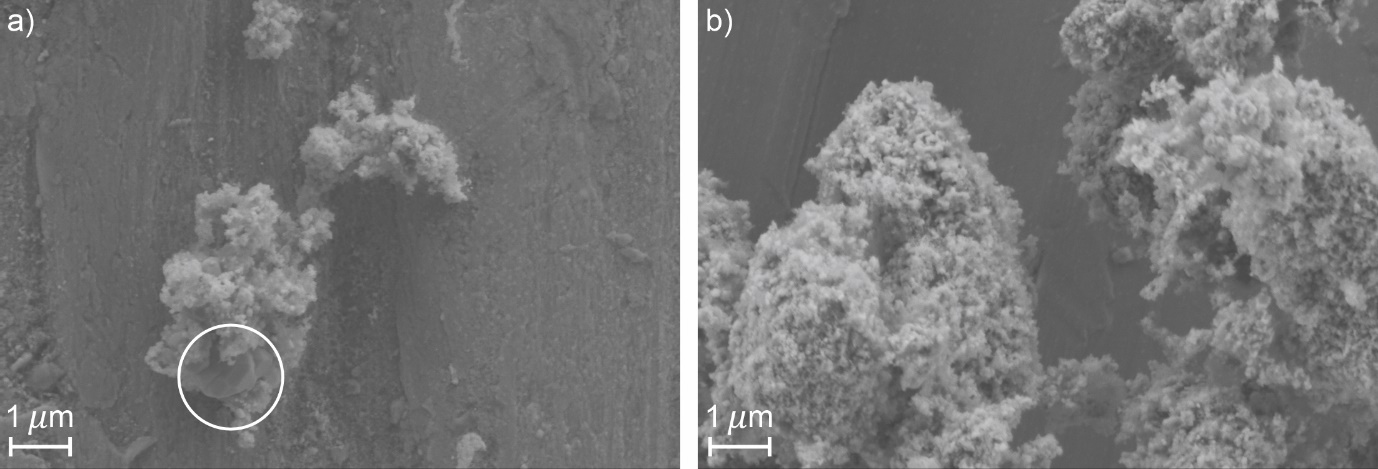


Supplementary Figure 3: SEM micrographs after high-temperature treatment with different SFTR-precursor : sucrose ratios. SFTR-precursor : sucrose = 1 : 0.2 (a) and SFTR-precursor : sucrose = 1 : 0.35 (b).

**1.4 BET surface area and pore size distribution**

**
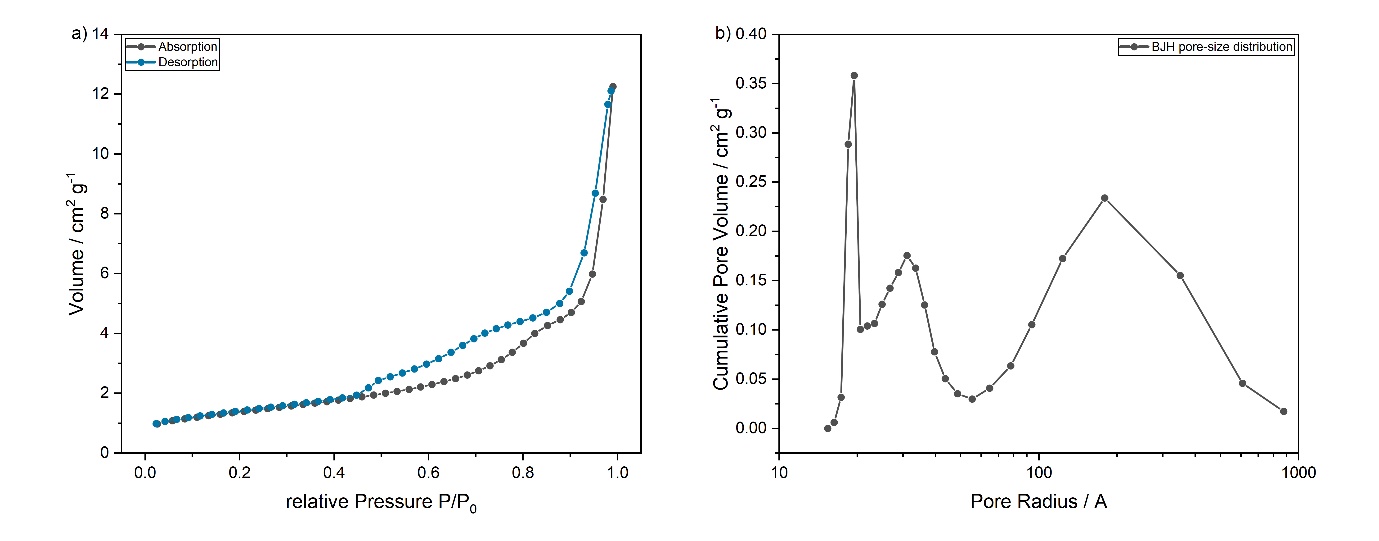
**

Supplementary Figure 4: N_2_-physisorption isotherms (a) and Barrett-Joyner-Halenda (BJH) pore-size distribution for NVP-SFTR.

**1.5 *in-situ* high-temperature XRD**


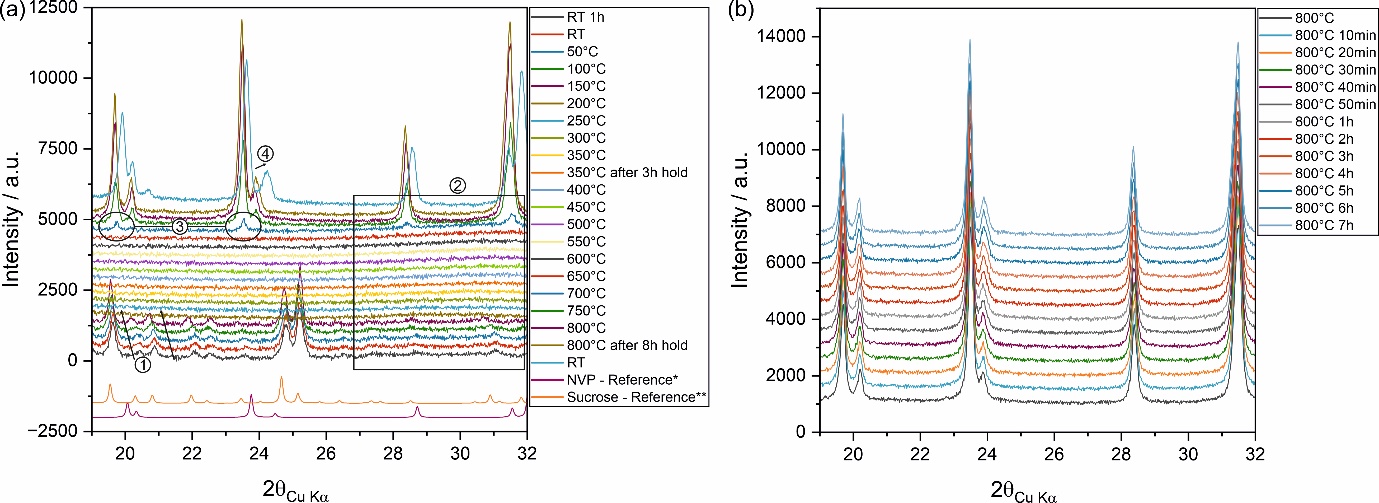


Supplementary Figure 5: Rainbow plot of selected patterns (a) and during the 800°C hold step (b) during in-situ XRD. The marked areas in (a) are described in the text. (1) Thermal expansion of the sucrose unit cell due to heating. (2) the amorphous bump, which disappears during the crystallization. (3) The onset temperature of the crystallization process between 650°C and 700°C. (4) the lattice contraction during cooling. The slight offset with respect to the references is due to expansion/contraction of the pelletized sample during the reaction.

**1.6 TGA-DSC analysis during the high-temperature treatment step**


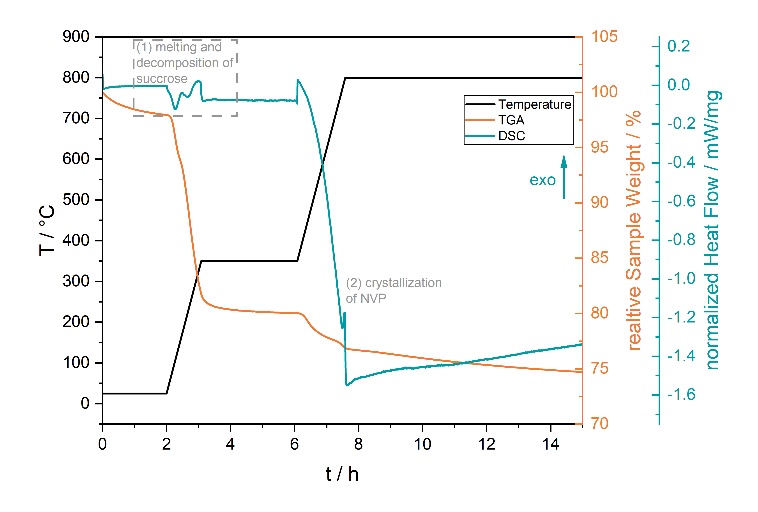


Supplementary Figure 6: TGA-DSC analysis during the high-temperature treatment step of NVP-SFTR.

**1.7 Morphology of NVP-hydro and NVP-solvo**


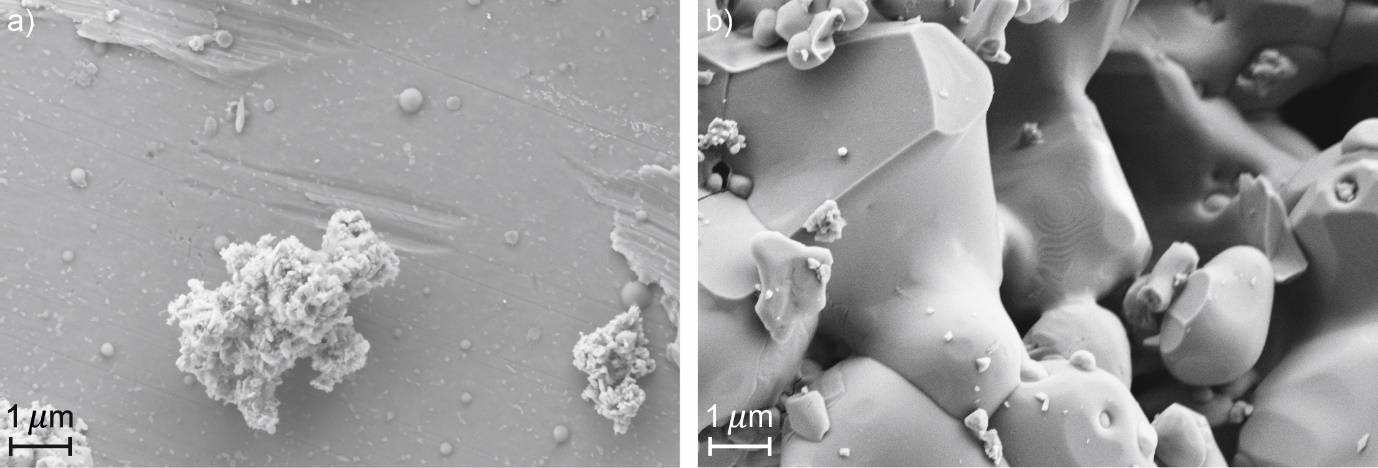


Supplementary Figure 7: SEM micrographs of NVP-solvo (a) and NVP-hydro (b) after high-temperature treatment (NVP-precursor : sucrose = 1 : 0.35)

**1.8 batch-to-batch variability**


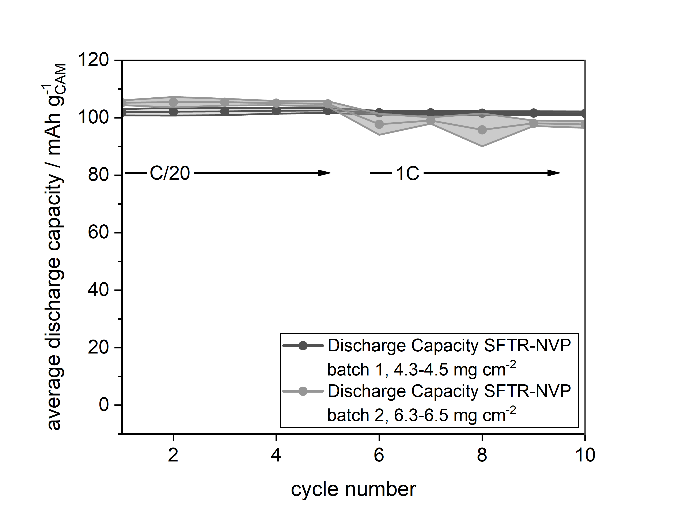


Supplementary Figure 8: batch-to-batch variability of averaged discharge capacities of two batches produced under identical settings (at different areal loadings). Between the two batches, the reactor was running for a total time of 2 days with multiple shutdowns (cooling and re-heating), using the same n-dodecane carrier oil. No significant n-dodecane degradation or loss was observed.
